# Analysis of partner of inscuteable (mPins) expression in the developing mouse eye

**Published:** 2008-12-31

**Authors:** B. Raji, A. Dansault, V. Vieira, G. de la Houssaye, E. Lacassagne, A. Kobetz, L. Arbogast, J.L. Dufier, J.B. Blumer, M. Menasche, M. Abitbol

**Affiliations:** 1 Université Paris-Descartes, Centre de Recherche Thérapeutique en Ophtalmologie de la Faculté de Médecine Paris-Descartes, Equipe d’accueil n°2502 du Ministère de la Recherche et de l’Enseignement Supérieur, AP-HP, CHU Necker-Enfants Malades, Paris, France; 2 Service d'Ophtalmologie, Hôpital Necker-Enfants Malades, Paris, France; 3 Department of Cell and Molecular Pharmacology, Medical University of South Carolina, Charleston, SC

## Abstract

**Purpose:**

Asymmetric cell division (ACD) is the fundamental mechanism underlying the generation of cellular diversity in invertebrates and vertebrates. During *Drosophila* neuroblast division, this process involves stabilization of the apical complex and interaction between the Inscuteable (Insc) and Partner of inscuteable (Pins) proteins. Both cell-intrinsic factors and cell–cell interactions seem to contribute to cell fate decisions in the retina. The Pins protein is known to play a major role in the asymmetric segregation of cell fate determinants during development of the central nervous system in general, but its role in asymmetric cell divisions and retinoblast cell fate has never been explored. The primary aim of this study was to determine the spatial distribution and time course of mouse homolog of *Drosophila* Partner of Inscuteable (mPins) expression in the developing and adult mouse eye.

**Methods:**

The expression pattern of *mPins* was studied in the mouse eye from embryonic (E) stage E11.5 until adulthood, by semiquantitative RT–PCR, in situ hybridization, and immunohistochemistry. In addition, variations in mRNA and protein levels for mPins were analyzed in the developing postnatal and adult lens, by semiquantitative RT–PCR, western blot analysis, in situ hybridization, and immunohistochemistry.

**Results:**

We detected *mPins* mRNA at early stages of mouse embryonic eye development, particularly in the neuroblastic layer. In early postnatal development, *mPins* mRNA was still detected in the neuroblastic layer, but also began to be detectable in the ganglion cell layer. Thereafter, *mPins* mRNA was found throughout the retina. This pattern was maintained in differentiated adult retina. Immunohistochemical studies showed that mPins protein was present in the neuroblastic layer and the ganglion cell layer during the early stages of postnatal retinal development. At these stages, mPins protein was colocalized with Numb protein, a marker of the ACD. At later postnatal stages, mPins protein was present in all retinal nuclear layers and in the inner plexiform layer. It continued to be detected in these layers in the differentiated retina; the outer plexiform layer and the photoreceptor inner segments also began to display positive immunostaining for mPins. In the adult retina, mPins was also detected in the retinal pigment epithelium and choroidal melanocytes. Throughout development, mPins protein was detected in nonretinal tissues, including the cornea, ciliary body, and lens. We focused our attention on lens development and showed that mPins protein was first detected at E14.5. The most striking results obtained concerned the lens, in which mPins protein distribution switched from the anterior to the posterior region of the lens during embryonic development. Interestingly, in the postnatal and adult lens, mPins protein was detected in all lens cells and fibers.

**Conclusions:**

We provide the first demonstration that mPins protein is expressed from embryonic stages until adulthood in the mouse eye. These results suggest that mPins plays important roles in eye development. This work provides preliminary evidence strongly supporting a role for mPins in the asymmetric division of retinoblasts, and in the structure and functions of adult mouse retina. However, the link between the presence of mPins in different ocular compartments and the possible occurrence of asymmetric cell divisions in these compartments remains to be clarified. Further studies are required to elucidate the in vitro and in vivo functions of mPins in the developing and adult human eye.

## Introduction

Cell proliferation and cell differentiation are fundamental processes in invertebrate and vertebrate development. They involve crucial events, such as cell polarization, segregation and localization of cell fate determinants, mitotic spindle orientation, and symmetric or asymmetric cell divisions. The establishment and maintenance of cell polarization are extremely important for epithelial cells and neurons, and for several other cell types. Three different groups of proteins have emerged as the key players in both epithelial cell and neuronal polarization: 1) the PAR proteins, 2) the CRB, Stardust and Patj (PALS1 and PATJ in mammals) proteins; and 3) a set of proteins including Scribble (Scrb), Discs-large (Dlg), and Lethal-giant-larvae (Lgl) [1]. Elegant experiments have shown that these 3 sets of proteins involved in cell polarization interact genetically to define the apical and basolateral surfaces of epithelial cells in *Drosophila* [2,3]. A large number of studies performed in *Drosophila* over the past 15 years have demonstrated strong associations between certain PAR proteins and the occurrence of asymmetric cell divisions during the development of the central (CNS) and peripheral nervous systems [4]. An example is provided by the development of the abdominal segment of the ventral nerve cord in the embryonic CNS in *Drosophila*. Each segment consists of about 700 neurons and 60 glial cells with different fates and morphologies, all derived from progenitor cells called neuroblasts (NBs). A diversity of cell fates is generated from a single precursor cell through programmed asymmetric cell division. During the asymmetric division of *Drosophila* NBs and sensory organ precursor cells, 2 different protein complexes have been shown to be necessary and to play different main roles: the mouse homolog of *Drosophila* Partner of Inscuteable (mPins)/Gαi complex is principally involved in spindle orientation (metaphase NBs align their spindles perpendicular to the epithelium layer) [5–8], whereas the PAR complex appears to be involved in the basal localization of cell-fate determinants [9–11]. PAR complex function requires 2 cortical tumor suppressors: Dlg and Lgl [12–14]. Dlg and Lgl are primarily involved in localizing basal proteins and have only a mild effect on the increasing formation of apical proteins [12,15]. However, in *Drosophila*, Pins interacts with Dlg and is a key protein in the Frizzled signaling pathway regulating the establishment of cell polarity and asymmetric cell division [16].

Two Pins homologs have been characterized in vertebrates: AGS-3 (activator of G protein signaling), which is found only in certain tissues, and Leu-Gly-Asn repeat-enriched protein (LGN), which is ubiquitous and has a sequence more similar to that of *Drosophila* Pins [16]. The mouse homolog of *Drosophila* Pins (mPins), also called LGN, has been identified [16]. The mPins protein has a similar amino acid sequence and similar functional domains to the *Drosophila* Pins protein, along its entire length [16]. *Pins* encodes a protein with 7 tetratricopeptide repeats (TPR) in its N-terminal region and 3 GoLoco motifs at its C-terminal region. TPR motifs are involved in protein–protein interactions [17], and are, in particular, responsible for interaction with the asymmetric localization domain of Inscuteable (Insc) [8]. GoLoco motifs bind to the Gαi subunit of heterotrimeric G proteins [5,6]. Deletion analysis of Pins has shown that, as in *Drosophila* [18], the C-terminal GoLoco-containing region specifies membrane targeting, whereas the N-terminal TPR further refines localization to the apical cortex [19]. In *Drosophila*, the Pins, Gαi, and Insc proteins form a complex. Pins and Insc are dependent on each other for apical asymmetric localization in delaminated NBs [6]. This leads to Pins/Gαi recruitment to the apical cortex in NBs. Pins activates Gαi, and this polarized activation of Gαi attracts 1 of the 2 spindle poles, thereby inducing reorientation of the mitotic spindle during asymmetric cell division (ACD) [20]. This Pins/Gαi/Insc complex appears to be conserved in mammals [6,21]. Pins is expressed in many mouse tissues, but its distribution in the CNS correlates with zones containing proliferative cells [19]. Mouse Pins has been shown to be a functional homolog of *Drosophila* Pins, able to display asymmetric localization and to substitute for Pins function in *Drosophila* neuroblasts [19]. Indeed, mammalian Pins physically interacts with the asymmetric localization domain of Insc through its TPR (TPR3-TPR7). Insc function is conserved in mammals and is required for correct orientation of the mitotic spindle in precursor cells of the rat retina [21]. These data suggest that the same Pins/Gαi/Insc complex may be involved in spindle reorientation and may lead to the specification of different cell types in the developing mammalian retina.

During development of the mammalian nervous system, a small number of progenitor cells gives rise to a huge variety of neurons and glial cells. ACD makes a significant contribution to this neuronal diversity. It has been demonstrated that ACD also contributes to the neuronal diversity of the retina, which is actually much greater than initially predicted by Ramon y Cajal [22] and most neuroscientists. Cell differentiation begins in the central part of the retina [23–25]. Cell divisions that give rise to differentiating cells initially occur only in the central retina. During the same period, cell divisions in the peripheral retina are exclusively symmetric and play an essential role in increasing the pool of progenitor cells [26]. There is evidence that both cell-intrinsic factors and cell–cell interactions contribute to cell fate decisions in the retina [27–30], but the relative importance and molecular bases of these elements remain unknown.

In summary, the mammalian retina displays a huge cell diversity and ACD has been shown to occur in rat [31,32] and chick [33] retina. Based on these data and the crucial role of Pins in asymmetric cell division in *Drosophila* neuroblasts [8], we focused this study on the determination of temporal and spatial expression patterns of the mouse homolog of *Drosophila Pins* (*mPins)* in the developing mouse eye, to determine what potential roles mPins could play in the development of the diverse mouse ocular compartments. We also determined mPins expression patterns both in term of mRNAs and proteins in the adult mouse retina to provide novel information concerning possible roles of mPins in adult mouse retinal physiologic functions.

## Methods

### Animals

All animals were handled in accordance with the Association for Research in Vision and Ophthalmology (ARVO) statement for the use of animals in ophthalmic and vision research. C57Bl6/j mice were kept at 21 °C, on a 12 h:12 h light-dark cycle, and given food and water ad libitum. C57Bl6/j mice, used for the preparation of tissue RNA extracts or tissue sections, were obtained from Charles River (L'arbresle, France). The date of conception was established by the presence of a vaginal plug in the dam and recorded as E0 (embryonic day 0). The day of birth was designated as P0 (postnatal day 0).

### Tissue preparation

The mouse embryos were microdissected from the whole trophoblast under a dissecting microscope. Microdissected embryos were placed on the surface of hard plastic cups filled with optimal cutting temperature (OCT) medium (Tissue Tek; Bayer Diagnostic, Puteaux, France). The lower surface of the cups was delicately isolated at the surface of a progressively refrigerating isopentane solution. The cups remained at the surface of the refrigerating isopentane solution until a temperature of −30 °C was reached. The specimens were subsequently frozen in powdered dry ice for 15 min and then stored at −80 °C until use. Cryostat sections (14 μm) were mounted on slides coated with 2% 3-aminopropyl-triethoxylane in acetone. Sections were fixed by incubation for 30 min in 2% paraformaldehyde in 0.1 M phosphate buffer (pH 7.4), rinsed once in phosphate-buffered saline (PBS; 137 mM NaCl, 2.7 mM KCl, 4.2 mM Na_2_HPO_4,_ and 1.47 mM KH_2_PO_4_ and adjusted pH to 7.4), rinsed briefly in water and dehydrated through a series of ethanol solutions of increasing concentration. Sections were then allowed to dry in air and stored at −80 °C. This procedure was used to preserve mRNAs in embryonic and fetal tissues.

E12.5–E18.5 mouse embryos and immature animals (from P1 to P12), as well as 30-day-old (P30) and 2-month-old (2M) mice, were killed by CO_2_ asphyxiation. Eyes were rapidly removed and fixed by incubation for at least 36 h in 4% PFA at 4 °C. They were then embedded in paraffin and 5 µm sections were cut with a microtome (HM355; Microm: Walldorf, Germany), mounted on glass slides (Superfrost Plus; Fisher Scientific, Illkirch, France), dried overnight at 37 °C and stored at room temperature until use.

### RNA extraction and reverse transcription-polymerase chain reaction

Total RNA was extracted from mouse eyes at different postnatal ages (P0, P8, P15, P21, and 2M), from lenses at different stages (P0, P14, P16, and 2M), and from the retina at 2M, using Trizol reagent (Invitrogen, Cergy-Pontoise, France) according to the manufacturer’s instructions. Next, 1 μg total RNA was reverse-transcribed using an oligodT primer with SuperScript II Rnase H Reverse Transcriptase (Invitrogen) in a total reaction volume of 20 μl. For semiquantitative PCR, the number of cycles and annealing temperature were optimized (data not shown). The PCR reactions were systematically optimized by testing serial dilutions of the amount of DNA templates, variable concentrations of the specific PCR primers, variable MgCl_2_ concentrations and variable annealing temperatures to obtain a specific and unique band of the appropriate expected size for each targeted co-amplified cDNA product. Each amplified product was subsequently checked systematically by automated sequencing to verify its molecular identity. We checked systematically all the adequate parameters to be sure that the number of cycles required for obtaining unique co-amplified bands was in the range of values comprised between 20 and 30 PCR cycles. This procedure provided us the guarantee that we were still in the linear phase of the exponential curve of PCR amplification and, thus, that we had not yet reached the plateau phase. The cyclophilin gene was coamplified with the target gene as an internal control for comparison.

We then amplified 1 μl of reverse-transcription product by PCR in a 10 μl reaction volume containing 2.5 µl of the 10 mM primer mixture, 0.5 U *Taq* DNA polymerase (Invitrogen), 1 µl 10X PCR buffer, 1 mM MgCl_2_, and 0.2 mM dNTP (Promega, Charbonnières-les-bains, France).

The *mPins* primers (Table 1) were designed to amplify a 473 bp fragment. The cyclophilin primers (Table 1) were designed to amplify a 311 bp fragment. All primers were synthesized by Invitrogen. The PCR conditions were as follows: mPins: 94 °C for 3 min, 30 cycles of 94 °C for 1 min, 58 °C for 1 min, and 72 °C for 1 min, with a final extension step at 72 °C for 7 min; Cyclophilin: 94 °C for 1 min, 25 cycles of 94 °C for 30 s, 55 °C for 30 s, and 72 °C for 1 min, with a final extension step at 72 °C for 7 min.

**Table 1:** Sequences of the primers of *mPins* and *Cyclophilin* cDNA fragments.

**Primers**	**Sequence (5′-3′)**
mPins-F	TACTAACCGGACAGTGCT
mPins-R	GGCAACACACTATCGCTTCA
cyclo-F	TGGTCAACCCCACCGTGTTCTTCG
cyclo-R	TCCAGCATTTGCCATGGACAAGA
mPins-AS probe	AAATGACACGCCAGCGCGGCAGTCCCCTGATTTACATAGACGTTCTCCTTCCAAGGCCAG
mPins-S probe	CTGGCCTTGGAAGGAGAACGTCTATGTAAATCAGGGGACTGCCGC GCTGGCGTGTCATTT
Scramble probe	ATCGTCAGCTGAGATCAATAATGGCCCCGGTTAGAGCTCTACTGCGATAATGGCTTGCCA

The nucleotidic sequences of the primers allowing the amplification of mPins and Cyclophilin cDNA fragments are showh in the four upper panels of the table; whereas the oligonuclodidic squences of the 60 mer probes used for isotopic in situ hybridizatiuon experiments are shown in the three lower panels of the table. The first 60 mer oligoprobe corresponds to the antisense probe recognizing the mPins mRNA, The second mer oligoprobe corresponds to the sense probe which serves as a negative control. It does not recognize any mRNA. The Scramble probe serves also as a control probe providing a further garantee of the specificity of our in situ hybridization protocol.

The PCR amplification products were analyzed by electrophoresis in a 2% agarose gel and visualized by ethidium bromide staining and transillumination under UV light, using a Syngene device (Ozyme, Saint-Quentin-en-Yvelines, France) and appropriate software to quantify the intensity of the bands observed. Each experiment was performed 3 times.

### Statistical analysis

All results are expressed as the mean±SD. The results were compared by ANOVA (ANOVA) and Student’s *t*-test. A p<0.001 was considered statistically significant.

### DNA probes for radioactive in situ hybridization

The mPins 60 mer oligonucleotide probes were synthesized and purified by Eurogentec (Angers, France). The oligonucleotides were 3′-end labeled with [^35^S]dATP (PerkinElmer, Courtabeuf, France), using 15 U/ml terminal deoxyribonucleotidyl transferase (Invitrogen-Gibco), to a specific activity of approximately 7×10^8^ cpm/mg, as previously described [34]. The protocol of labeling of the probe was the following one: to 1 μl containing 200 ng of the the oligonucleotidic 60 mer probe, that had to be labeled, were added 5.5 μl of [^35^S]dATP (PerkinElmer,Courtabeuf, France), 6.5 μl 15 U/ml terminal deoxyribonucleotidyl transferase (Invitrogen-Gibco) and 12 μl of water. The ensemble of the reagents were gently mixed by pipetting slowly the solution. Then the eppendorf tube containing the 25 μl of solution was dipped partially in a water incubator the temperature of which had previously been set at 37 °C at least one hour before starting the labeling reaction. The enzymatic reaction was left proceeding for 5 min. Then the eppendorf tube was put in ice at + 4 °C to stop the catalytic process. Each oligonucleotidic probe was purified on biospin P30 columns twice before use (BioRad, Ivry-sur-Seine, France)

The *mPins* probes were designed based on the mouse *Pins* cDNA sequence (AY081187). The sense and scramble probes were used as negative controls. The scramble probe contained nucleotides randomly chosen by an appropriate algorithm. When the scramble probe was compared with the entire GenBank and EMBL nucleotide sequence databases, no matching sequence could be retrieved. The sequences of the probes were described in Table 1.

### Radioactive in situ hybridization procedure

In situ hybridization was performed on frozen mouse embryo sections prepared as described in the previous section. The hybridization cocktail contained 50% formamide, 4X standard saline citrate (SSC), 1X Denhardt’s solution, 0.25 mg/ml yeast tRNA, 0.25 mg/ml sheared herring sperm DNA, 0.25 mg/ml poly(A)^+^, 10% dextran sulfate (Sigma, Saint-Quentin Fallavier, France), 100 mmol DTT, and one of the [^35^S]dATP-labeled probes, at a concentration of 6×10^5^ cpm/100 µl of final hybridization mixture. We applied 100 µl of hybridization solution to each section. The sections were covered with a Parafilm coverslip and incubated in a humidified chamber at 43 °C for 20 h. They were then washed twice for 15 min in 1X SSC supplemented with 10 mM DTT at 55 °C, twice for 15 min in 0.5X SSC supplemented with 10 mM DTT at 55 °C, and once for 15 min in 0.5X SSC supplemented with 10 mM DTT at room temperature. The sections were then dipped in water, dehydrated by immersion in a series of ethanol solutions of increasing concentration and placed against X-ray film (Hyperfilm Betamax; Amersham, Orsay, France) for 1 week. They were then treated with photographic emulsion (NTB2; Eastman Kodak, Rochester, NY) and incubated for 2 months at 4 °C. Sections were developed, counterstained with 0.2% toluidine blue in 0.2 M sodium acetate, pH 4.3, covered with a coverslip, and examined under bright- or dark-field illumination. Both the bright- and dark-field images were acquired with a charge-coupled device (CCD) camera (Nikon, Tokyo, Japan) connected to a computer Image J software.

### Probes for non isotopic in situ hybridization

Antisense and sense (DIG)-labeled riboprobes were synthesized with T7 RNA polymerase, from *mPins* fragment PCR products. These were labeled with a 10X DIG RNA labeling kit (Promega).

### PCR in situ hybridization procedure

PCR in situ hybridization experiments were performed on deparaffinized, rehydrated 5 µm eye sections from C57Bl6/j animals. Sections were incubated overnight at 65 °C with the probes, and washed with 1X Stringent Wash Concentrate (Dako, Trappes, France), according to the manufacturer’s instructions. Sections were then incubated for 1 h at room temperature with a conjugate anti-DIG–AP antibody (11093274; Roche, Mannheim, Germany) and rinsed in 1X PBS. Tissue sections were then incubated with nitroblue tetrazolium/5-bromo-4-chloro-3-indolyl-phosphate (NBT/BCIP) for 30 min in the dark. Slides were mounted in Aquatex (Merck, Darmstadt, Germany) and examined under a Leica DMRB microscope (Leica, Rueil-Malmaison, France). We applied the same quantity of probe to each slide and treated all slides in a single experiment to ensure that they could be compared.

### Immunohistochemistry

Postnatal and adult paraffin-embedded eye sections (see Tissue preparation) were deparaffinized by incubation in xylene and rehydrated through a graded series of alcohol solutions. Frozen embryonic tissue sections were treated with acetone. All sections were labeled with the detection kit (ChemMate; Dako), according to the manufacturer’s instructions. Three different primary antibodies were used to detect mPins protein: 1:200 rabbit anti-Pins antibody (provided by Xiaohang Yang, Institute of Molecular and Cell Biology, Singapore) [19], and two rabbit anti-Pins antibodies (1:300 LGN Ser^417^-Lys^449^; 1:150 LGN-Cterm; both provided by Joe B. Blumer, Department of Cell and Molecular Pharmacology, Medical University of South Carolina, Charleston, SC) [35]. The secondary antibody used was a biotinylated antibody (ChemMate detection kit; Dako), with diaminobenzidine (DAB) as the substrate. The DAB-stained tissue sections were counterstained with 3% methyl green (Sigma, Saint-Quentin Fallavier France). The slides were then mounted in Eukitt (O. Kindler, Freiburg, Germany) and examined under a light microscope.

### Immunohistofluorescence

The paraffin was removed from murine eye sections (see Tissue preparation) by incubation in xylene, and the tissue sections were then rehydrated by incubation in a graded series of ethanol solutions. Sections were then incubated overnight at 4 °C with 1:200 rabbit anti-Pins antibody [19] or 1:300 guinea pig anti-Numb antibody (provided by Weimin Zhong, Department of Molecular, Cellular, and Developmental Biology, Yale University, New Haven, CT) [36]. Sections were washed in 1X PBS and incubated for 2 h at room temperature in a dark chamber with appropriate secondary antibodies: 1:300 Alexa Fluor 488-conjugated anti-rabbit (A21206), 1:300 Alexa Fluor 633-conjugated anti-rabbit (A21070) and 1:300 Alexa Fluor 488-conjugated anti-guinea pig secondary antibodies (A11073). All secondary antibodies were purchased from Invitrogen-Gibco. Following incubation with the secondary antibody, the sections were washed with 1X PBS in the dark and mounted in Dako Cytomation fluorescent mounting medium. Sections were stored at 4 °C until viewing on a confocal microscope.

### Colocalization measures

Colocalization was defined as the presence of 2 stains so close together in the studied tissue that they could not be resolved optically. The extent of colocalization of the 2 labels (mPins and Numb) was measured using the “Coloc” module of Imaris 6.1.2, 32-bit version (Bitplane AG, Saint Paul, MN). Each confocal section consists of an array of square elements called pixels. A voxel is defined from a pixel as a prism in which the base is the pixel and the height is the thickness of the confocal section. Imaris colocalization analyzes the confocal stack by measuring the intensity of each label in each voxel. The program uses a determined threshold for each of the two labels (10 on the 0–255 scale of pixel intensity). Voxels with intensities above this threshold are considered to be above the background. A voxel is defined as displaying colocalization when the intensities of both labels are above their respective thresholds. The extent of colocalization is expressed in terms of two measures: 1) “Percentage of material colocalized,” taking into account the number of voxels displaying colocalization and the intensities of the two labels in each voxel; “material” takes into account both the number of voxels and their intensities; and 2) “Pearson correlation coefficient” calculated for voxels, giving values of between +1 and −1, with positive values indicating a direct correlation, negative values indicating an inverse correlation, and values near 0 indicating no correlation. This measure is more stringent than the “Percentage of material colocalized” because it also requires the intensities of the 2 labels to vary together.

### Western blot

Total proteins were extracted separately from the lens at P0, P14, P16, and 2M, from the retina and total eye of adult C57Bl/6J mice, using an extraction reagent (TRIzol; Invitrogen-Gibco) according to the manufacturer's instructions. Protein concentrations were determined with the Bradford protein assay. We mixed 50 µg of protein from each sample 1:1 with a loading buffer, pH 6.8, that contained 60 mM Tris, 10% glycerol, 2% sodium dodecyl sulfate, 5% 2-betamercaptoethanol, and 0.01% bromophenol blue. We then boiled the sample for 5 min and subjected it to electrophoresis in a 10% polyacrylamide gel containing SDS. Proteins were transferred onto a nitrocellulose membrane (Bio-Rad Laboratories, Hercules, CA), and nonspecific binding was blocked by incubation with 5% skim milk for 1 h. Membranes were then incubated for 2 h with either 1:1,000 rabbit anti-Pins antibody (provided by Xiaohang Yang, Institute of Molecular and Cell Biology, Singapore) [19], or two rabbit anti-Pins antibodies (1:1,500 LGN Ser^417^-Lys^449^; 1:1,000 LGN-Cterm; both provided by Joe B. Blumer, Department of Cell and Molecular Pharmacology, Medical University of South Carolina, Charleston, SC) [35] and 1:1,000 goat anti-β-actin antibody (C11; Santa Cruz Biotechnology, Santa Cruz, CA). Membranes were washed in 0.5% Tween-20 in PBS and incubated for 2 h with the appropriate horseradish peroxidase-conjugated secondary antibody: anti-rabbit (1:5,000 SC2030, Santa Cruz) or anti-goat (1:5,000 SC2033; Santa Cruz). Proteins were then detected by chemiluminescence assays (ECL; PerkinElmer Life and Analytical Sciences, Inc.).

## Results

### *mPins* mRNA levels in postnatal and adult mouse eye

**Figure 1:**
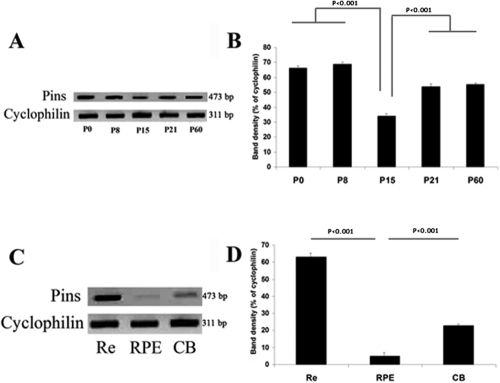
*mPins* mRNA levels in mouse eye during development and adulthood. **A** and **C** show semiquantitative RT–PCR determinations of the relative amounts of *mPins* mRNA in mouse whole eye at P0, P8, P15, P21, and P60 (**A**); and in neuroretina (Re), in the retinal pigment epithelium (RPE), and in the ciliary body (CB) of adult mouse (**C**). The *cyclophilin* mRNA was used as an internal control. The 473 and 311 bp bands correspond to the RT–PCR products for *mPins* and *cyclophilin*, respectively. **B** and **D** show densitometric analysis of the intensity of the PCR bands corresponding to **A** and **C**, respectively. The RNA blot images are cropped very tight. However the lanes were otherwise indeed free of signal and the results of densitrometric analysis were not affected by the cropping. The relative levels are calculated as the ratio of intensity of the *mPins* band to that of the *cyclophilin* band. The intensity of the *mPins* band does not seem to change during postnatal eye development, with the exception of the lower intensity of the *mPins* band at P15 (p<0.001; **A** and **B**). *mPins* mRNA levels are higher in the retina than in the RPE and CB (n=5; **C** and **D**). Error bars indicate the SEM.

*mPins* expression in whole eye was evaluated by RT–PCR at several stages of mouse development. The PCR-amplified product obtained with *mPins*-specific primers produced clear bands of the predicted size, 473 bp. *mPins* mRNA was detected at all postnatal stages studied (Figure 1A). After normalization with respect to *cyclophilin* mRNA (internal control), *mPins* mRNA levels were found to remain roughly constant throughout eye development, except at P15, when a significant decrease was observed (p<0.001; Figure 1B).

We then studied *mPins* gene expression in various ocular tissues from adult mice eye (Figure 1C). *mPins* gene expression was detected in mouse neuroretina, retinal pigment epithelium (RPE) and choroid, and ciliary body (CB) extracts. Moreover, after normalization with respect to cyclophilin mRNA, *mPins* mRNA levels were found to be higher in the retina than in the CB and RPE and choroid (p<0.001; Figure 1D).

### Localization of *mPins* mRNA in embryonic, postnatal, and adult eye

**Figure 2:**
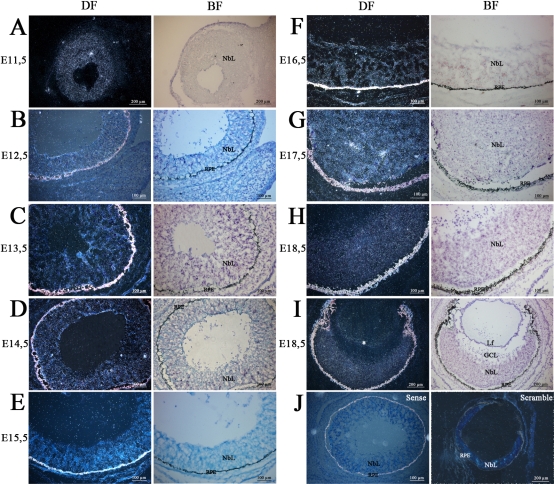
*mPins* mRNA localization in mouse embryo by radioactive in situ hybridization. *mPins* labeling with dark-field illumination (left) and the corresponding bright-field (right) are shown for stages E11.5 (**A**), E12.5 (**B**), E13.5 (**C**), E14.5 (**D**), E15.5 (**E**), E16.5 (**F**), E17.5 (**G**) and E18.5 (**H** and **I**). Dark-field negative controls using sense (**J**, left) and scramble (**J**, right) are also shown. *mPins* transcripts are detected in the neuroblastic layer (NbL) of the retina from E11.5 to E18.5 of embryonic development (**A**-**I**). The corresponding bright-field views confirm these observations (black grains). At E18.5, no significant signal is detected in the ganglion cell layer (**H**). At the same stages, we also observe a strong signal at the posterior face of the lens, corresponding to secondary fiber cells (**I**, asterisk). Negative controls using sense (**J**, left) and scramble (**J**, right) probes gave no significant signal.

We characterized the distribution of *mPins* mRNA in the mouse eye during embryonic development, by studying the pattern of *mPins* gene expression at various stages by isotopic in situ hybridization on longitudinally oriented frozen embryonic tissue sections. We analyzed *mPins* gene expression from stage E11.5, when the first retinal ganglion cells are established, until adulthood, when all retinal cell types are present. From stages E11.5 to E18.5, *mPins* expression was detected in the ocular neuroblastic layer (NbL; Figure 2A-I), which at this stage consists of cells in various states of commitment toward proliferation as well as differentiation. No significant *mPins* labeling could be observed in the ganglion cell layer (GCL). The developing RPE was autofluorescent, and we were not able to detect any in situ hybridization signal. At E18.5, a significant signal was observed exclusively in the posterior part of the lens, corresponding to secondary lens fibers (Figure 2I, asterisk). Before this stage, the signal in the lens seemed to be under the threshold of detectability. No significant signal was detected in any other structure of the eye at any of the stages studied (data not shown). From stage E11.5 to birth, *mPins* transcripts were consistently detected throughout the developing CNS (data not shown). The specificity of *mPins* mRNA detection was further confirmed by negative controls (Figure 2J), using sense (left) and scramble (right) probes, which gave no specific hybridization signal.

**Figure 3:**
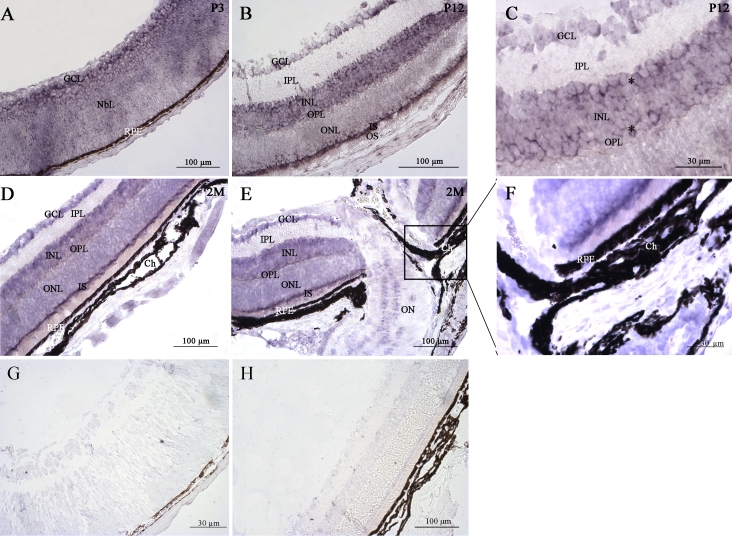
*mPins* mRNA localization in the postnatal and adult retina by in situ PCR hybridization. *mPins* labeling is shown in the postnatal retina at P3 (**A**) and P12 (**B**), and in the adult retina (**D** and **E**). **C** and **F** are higher magnifications of **B** and **E**, respectively. **G** and **H** show negative controls at P3 and in adult retina, respectively, using the mPins sense probe. At the P3 stage, significant *mPins* mRNA labeling is detected in the neuroblastic (NbL) and ganglion cell layers (GCL; **A**). In the retina, at the P12 stage, significant *mPins* mRNA is detected in the GCL, the inner nuclear layer (INL), the outer nuclear layer (ONL), and in the inner segments of the photoreceptors (IS; **B**). High magnification of retina at this stage shows that *mPins* mRNA labeling is more intense on either side of the INL (**C**, asterisks). No significant labeling is observed in either of the plexiform layers (**B** and **C**). A similar pattern of *mPins* expression is observed in the adult retina (**D** and **E**). *mPins* mRNA is detected in cell bodies on sections of the optic nerve (ON; **E**). Significant *mPins* labeling is also detected at this stage in the retinal pigment epithelium (RPE) and choroidal melanocytes (Ch; **F**).

We investigated the pattern of *mPins* gene expression in eye in more detail, by carrying out nonisotopic in situ hybridization with PCR-amplified probes labeled with digoxigenin on longitudinally oriented eye sections from mice at various postnatal stages and in adulthood. At the P3 stage, *mPins* mRNA was still detected in the neuroblastic layer, as during embryonic stages. However, significant labeling of *mPins* mRNA was also found in the GCL at this stage (Figure 3A), whereas no such labeling was observed in embryonic eyes. A similar pattern was observed at P5 (data not shown). At P12, *mPins* mRNA was observed in all retinal nuclear layers: the GCL, the inner and outer nuclear layers. Strong *mPins* mRNA labeling was also detected in the photoreceptor inner segments (IS; Figure 3B). High magnification of retinal tissue sections at this stage showed that the *mPins* mRNA signal was more intense on either side of the inner nuclear layer than in the middle of this layer (Figure 3C, asterisks). No significant labeling was observed in any plexiform layer (Figure 3B,C). A similar pattern of *mPins* gene expression was observed in the adult retina (Figure 3D,E). It is noteworthy that *mPins* mRNA was also detected in the RPE and choroidal melanocytes, but not in the choroidal vascular endothelial cells or vascular uveal endothelial cells (Figure 3F). We also detected *mPins* mRNA in cell bodies localized in the optic nerve (Figure 3E). The specificity of *mPins* mRNA detection was confirmed by the use of sense probes, which gave no specific hybridization signal (Figure 3G,H).

**Figure 4:**
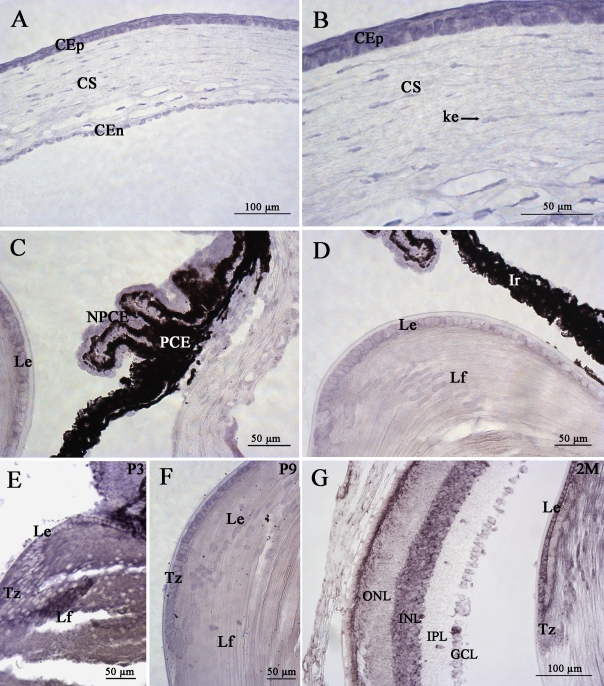
mPins mRNA localization in nonretinal structures by in situ hybridization. *mPins* mRNA localization in the adult cornea (**A** and **B**), the ciliary body (**C**), the iris (**D**) at 2 months (2M), and in the lens at P3 (**E**), P9 (**F**) and at 2M (**G**). In the cornea, *mPins* mRNA is observed in the epithelium (CEp), stroma (CS), and endothelium (CEn; **A**). A high magnification of **A** also shows *mPins* mRNA to be present in the stromal keratocytes (ke; **B**). Significant labeling was also detected in the nonpigmented ciliary epithelial cells (NPCE) of the ciliary body (**C**). No significant labeling is observed in the pigmented ciliary epithelial cells (PCE; **C**), and weak *mPins* mRNA labeling is detected in the iris (Ir; **D**). In the lens, *mPins* mRNA is observed in the epithelium (Le), transitional zone (Tz), and lens fibers (Lf) from early postnatal stages to adulthood (**D-G**).

We also investigated the distribution of *mPins* mRNA in other ocular structures. The *mPins* gene was widely expressed during postnatal development and in the adult eye at the 2 month post-natal stage (2M i.e adulthood). In the cornea, CB, and iris, *mPins* mRNA in situ hybridization signals were readily detectable at postnatal stages (data not shown) and in adult mice, in which the cellular distribution was identical. We therefore show only the in situ hybridization signals observed at the adult stage (Figure 4A-D). Strong labeling was observed in the corneal epithelial cells, endothelium, and stromal keratocytes (Figure 4A,B). In the CB, *mPins* mRNA was observed in the nonpigmented ciliary epithelial cells, but no significant labeling was detected in the pigmented ciliary epithelial cells (Figure 4C). Faint *mPins* labeling was also detected in the iris (Figure 4D). This faint labeling in the iris and the absence of detection in pigmented CB cells may result from the high density of melanin in these structures. From P3 to adulthood, *mPins* mRNA was also detected in the epithelial cells, transitional zone, and lens fiber cells (Figure 4D-G).

### mPins protein expression in developing and adult mouse retina

**Figure 5:**
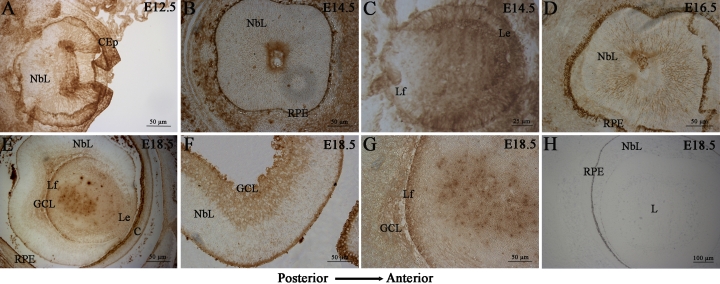
mPins protein localization in embryonic mouse retina by immunohistochemistry. mPins protein localization is shown in embryonic mouse retina at stages E12.5 (**A**), E14.5 (**B**), E16.5 (**D**), and E18.5 (**E** and **F**). mPins protein distribution is also shown in lens at E14.5 (**C**) and E18.5 (**G**). Orientation of mouse eye sections is specified below the figure. From E12.5 to E18.5, mPins protein is detected in neuroblastic layer (NbL; **A**, **B**, **D**, **E,** and **F**). Furthermore, mPins immunolabeling is stronger in the inner part of the NbL from E16.5 (**D-F**). At this stage, the mPins protein is also observed in the ganglion cell layer (GCL; **F**). The mPins protein is also found in the lens with a specific distribution depending on the stage (**C** and **G**). At the E14.5 stage (**C**), immunostaining is observed in the anterior region, whereas staining is observed only in the posterior region of the lens by E18.5 (**G**). Moreover, mPins protein is observed in the corneal epithelium, the presumptive cornea from E12.5 (**A** and **E**). **H** shows Diaminobenzidine (DAB) immunonegative control.

We investigated the overlap between the mPins protein and mRNA distributions by exploring protein levels in ocular tissue sections. Previous birth-dating studies have indicated that retinal histogenesis is normally completed by the end of the second week after birth (around P11) [37,38]. We investigated mPins protein distribution in embryonic eye tissue sections (Figure 5). mPins protein was observed in the neuroblastic layer from stages E12.5 to E18.5 (Figure 5A,B,D-F). mPins immunostaining was strongest in the inner part of the neuroblastic layer at later embryonic stages (Figure 5D-F). We also observed mPins immunolabeling in the GCL (Figure 5F). By contrast with the distribution of *mPins* mRNA, significant amounts of mPins protein were detected from E12.5 in the corneal epithelium, the presumptive cornea (Figure 5A,E). Moreover, mPins protein was also detected in the developing lens (Figure 5C,G). However, in this structure, mPins immunoreactivity seemed to have a specific distribution, depending on the embryonic stage studied. At the E14.5 stage, mPins cellular immunolabeling was found in the anterior region of the lens corresponding to the lens epithelium, whereas at the E18.5 stage, mPins protein was detected only in the posterior region corresponding to secondary lens fibers. No specific signal was detected in immunohistochemical negative control experiments (Figure 5H).

**Figure 6:**
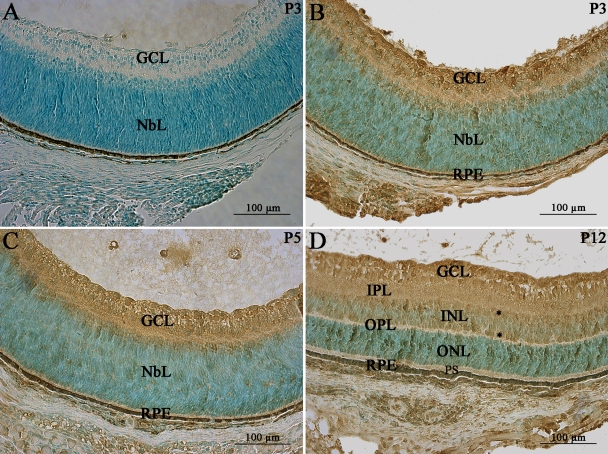
mPins protein localization in postnatal mouse retina by immunohistochemistry. mPins protein localization is shown in the developing mouse retina at postnatal stages P3 (**B**), P5 (**C**), and P12 (**D**). At stages P3 (**B**) and P5 (**C**), mPins protein is detected primarily in the ganglion cell layer (GCL) and the neuroblastic layer (NbL). In the retina at stage P12, mPins is observed in the GCL, the inner nuclear layer (INL), and the outer nuclear layer (ONL; **D**). Moreover, in the INL, mPins labeling was found to be more intense on either side of this layer than in its center (asterisks). Immunolabeling is also detected in the inner plexiform layer (IPL), but no labeling is observed in the outer plexiform layer (OPL) or in the photoreceptor segments (PS; **D**). **A** shows Diaminobenzidine (DAB) immunonegative control.

We also explored mPins protein distribution in postnatal retina from P3 to P12. At early stages (P3 and P5), mPins immunostaining was found in the GCL and neuroblastic layers (Figure 6B,C). At P12, mPins immunoreactivity was detected in all nuclear cell layers, including the GCL, the inner and outer nuclear layers.

Immunolabeling for mPins in the inner nuclear layer seemed to be more intense on either side of this layer than in the middle of this layer (asterisks). Moreover, mPins immunostaining was also observed in the inner plexiform layer, but not in the outer plexiform layer (Figure 6D). In control experiments, no specific immunolabeling was apparent when the primary anti-mPins antibody was omitted (Figure 6A).

**Figure 7:**
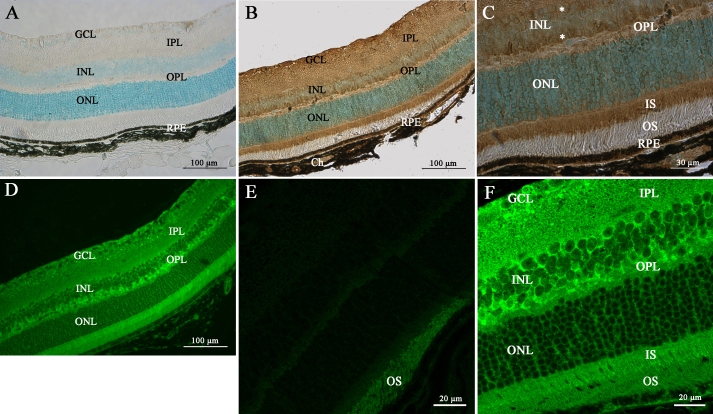
mPins protein localization in the adult mouse retina by immunohistochemistry. **A**-**C** show mPins protein localization by DAB chromogen immunochemistry. **A** shows the DAB-immunonegative control. **D**-**F** show confocal views of mPins protein localization by immunofluorescence; **E** shows the immunonegative control. mPins protein is detected in all retinal nuclear layers: the ganglion cell layer (GCL), the inner nuclear layer (INL), and the outer nuclear layer (ONL). The protein is also observed in the inner plexiform layer (IPL), with weak labeling detectable in the outer plexiform layer (OPL; **B** and **C**). mPins protein is also observed in the retinal pigment epithelium (RPE) and in the choroidal melanocytes (Ch; **C**). Higher magnification also shows strong immunolabeling in the inner segments of the photoreceptors (IS; **C**). Moreover, in the INL, mPins labeling is stronger on either side of the layer than in its center (**C**, asterisks). Confocal views confirm this distribution of protein and show the mPins protein to be present principally in the cellular membrane of the INL and ONL cells (**D** and **F**).

No immunolabeling was detected in control experiments (Figure 7A), whereas mPins protein was detected in all nuclear cell layers, including the GCL, the inner and outer nuclear layers in adult retina (Figure 7B). As described, at P12, mPins immunolabeling in the inner nuclear layer seemed to be more intense on either side of the layer than in the middle of the layer (asterisks; Figure 7C). mPins immunoreactivity was not restricted to the inner plexiform layer, with weak but significant mPins immunolabeling also observed in the outer plexiform layer (Figure 7B,C). Interestingly, by contrast to what was observed at earlier postnatal stages, strong mPins immunoreactivity was observed in the photoreceptor inner segments (IS) of the adult retina (Figure 7C). mPins immunostaining was unambiguously observed in the retinal pigment epithelium and choroidal melanocytes (Figure 7C). Confocal microscopy was performed to validate the distribution of mPins. The confocal microscopy results confirmed the detection of mPins protein in the retina. They also suggested that mPins immunolabeling might be associated principally with cell membranes or observed next to these cell membranes in the inner and outer nuclear layer. However, further studies with specific markers are required to confirm this hypothesis (Figure 7D,F). No specific signal was detected in immunohistochemical negative control experiments, in which only the autofluorescence of the IS was detected, as expected (Figure 7E).

### mPins protein expression in postnatal and adult mouse non retinal ocular structures

**Figure 8:**
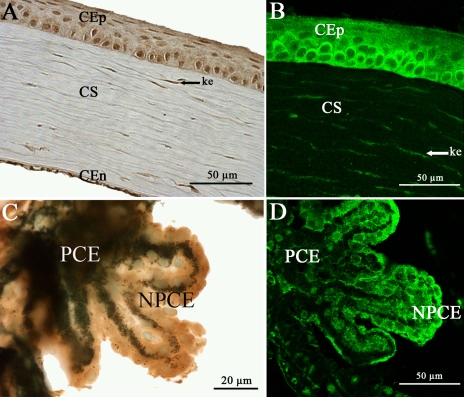
mPins protein localization in the adult mouse cornea and ciliary body by immunohistochemistry. **A** and **C** show localization of the mPins protein in the adult cornea and ciliary body. **B** and **D** are the corresponding confocal views. In the cornea, mPins protein is observed in the epithelium (CEp), stroma (CS), and endothelium (CEn; **A**). The confocal view (**B**) shows mPins localization in the stromal keratocytes (ke, arrows). In the ciliary body, mPins protein is detected in the nonpigmented ciliary epithelial cells (NPCE). No significant labeling is observed in the pigmented ciliary epithelial (PCE) cells (**C** and **D**).

We also explored the distribution of mPins protein in non retinal ocular structures. A high magnification of the cornea showed intense mPins immunolabeling in the corneal epithelial (CEp), and corneal endothelial cells (CEn). Strong mPins immunostaining was also detected in stromal keratocytes (Figure 8A,B). In the ciliary body, mPins immunoreactivity was observed in the nonpigmented ciliary epithelium (NPCE), but not in the pigmented ciliary epithelium (PCE; Figure 8C,D). The distribution of mPins in these structures was similar from early stages of postnatal development (data not shown) until adulthood.

**Figure 9:**
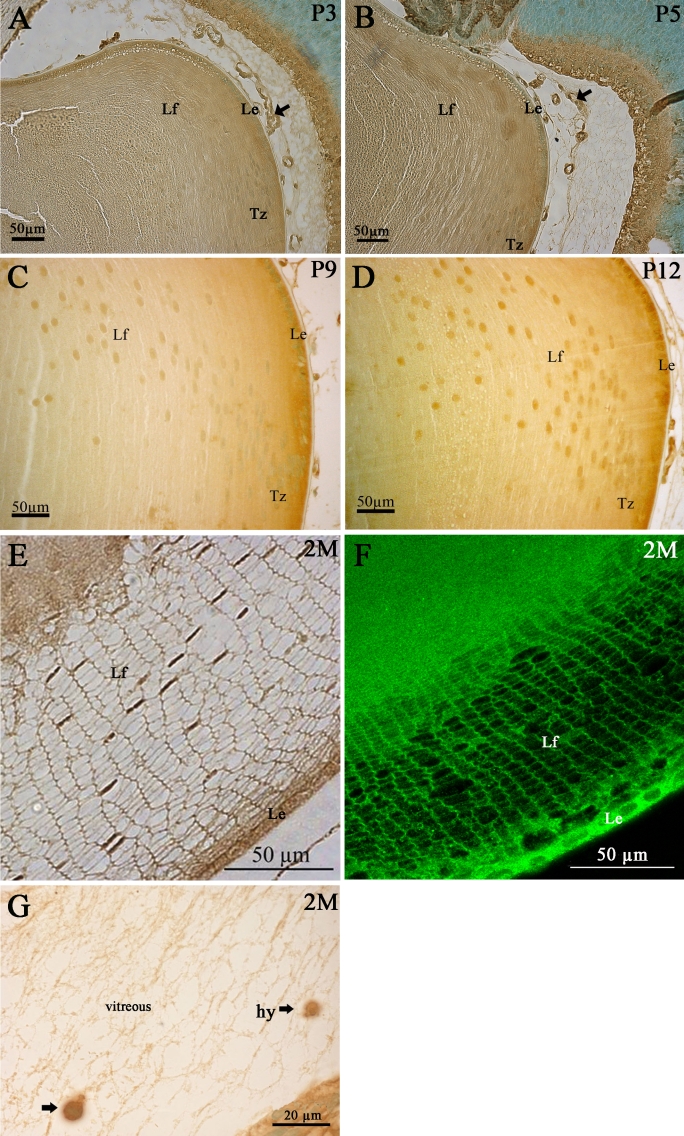
mPins protein localization in the adult mouse lens by immunohistochemistry. mPins protein localization is shown in the lens at postnatal stages P3 (**A**), P5 (**B**), P9 (**C**), and P12 (**D**), and in the adult lens (**E-G**). At the early postnatal stages, P3 and P5, diffuse labeling is observed in the lens. However, the mPins protein is detected in the tunica vasculosa (**A** and **B**, arrows). The mPins protein is found in the lens from later postnatal stage (P9) and is localized in the lens epithelial cells (Le), the lens fiber cells (Lf), and the transitional zone (Tz). This expression pattern is maintained at stage P12 (**C** and **D**) and throughout adulthood (**E** and **F**). Moreover, significant immunoreactivity is observed in the hyalocytes (hy) in the vitreous humor (**G**, arrows).

However, mPins protein expression differed between the early and late postnatal stages. At the P3 and P5 stages, the mPins protein seemed to be present in the lens but we were not able to determine any mPins immunopositive cell types due to diffuse staining (Figure 9A,B). At the P3 and P5 stages, mPins immunoreactivity was also detected in the endothelial cells of the tunica vasculosa lentis, around red and white blood cells (Figure 9A,B, arrows). By contrast, at P9 and P12, strong mPins immunoreactivity was observed in both lens epithelial cells and early differentiating secondary lens fiber cells, which were clearly mPins immunostained and could be clearly separated (Figure 9C,D). Moreover, at these stages, we also detected mPins immunoreactivity in the transitional zone (Figure 9C,D). This expression pattern was maintained in the adult lens (Figure 9E,F). At this stage, specific mPins immunoreactivity was clearly observed in the hyalocytes located within the vitreous (Figure 9G).

**Figure 10:**
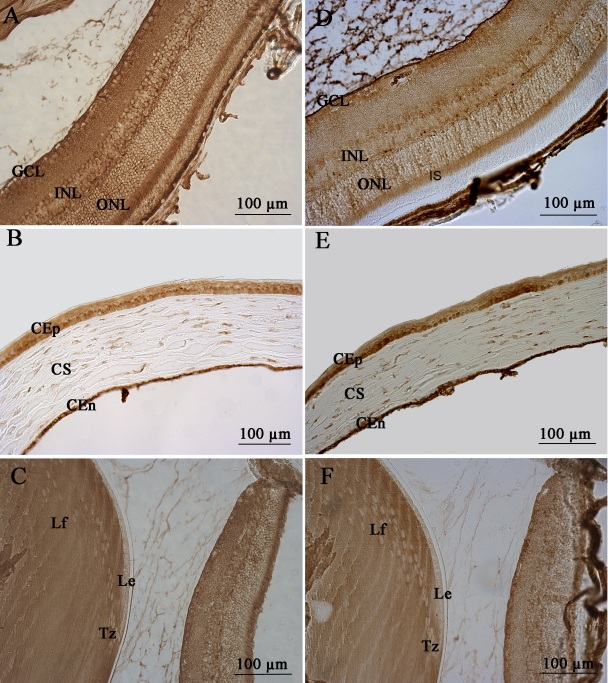
mPins protein localization in adult mouse ocular structures by immunohistochemistry with two different antibodies. **A**-**C** show the distribution of mPins protein as determined with an antibody against LGN (Ser^417^-Lys^449^) in adult retina (**A**), cornea (**B**), and lens (**C**). **D-F** show mPins protein localization with an antibody against LGN-Cterm in adult retina (**D**), cornea (**E**), and lens (**F**). mPins expression pattern was identical in retina, cornea, and lens with each of the different antibodies used.

We demonstrate here that mPins protein is indeed expressed in the retina and nonretinal ocular structures during development and in adulthood. These results were obtained with an antibody provided by Xiaohang Yang (Institute of Molecular and Cell Biology, Singapore) [19], but an identical pattern of mPins immunostaining was observed in all these structures with two antibodies recognizing different parts of the protein provided by Joe B. Blumer (Department of Cell and Molecular Pharmacology, Medical University of South Carolina, Charleston, SC) [35] (Figure 10). The identical immunostaining profiles, obtained with three different antibodies against different specific regions of the mPins protein and from different laboratories, provide strong evidence for the specificity of the immunostaining observed.

### mPins and Numb colocalization analysis

**Figure 11:**
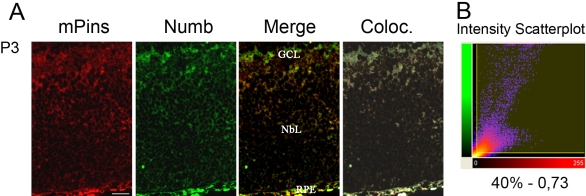
Colocalization of mPins and Numb proteins in undifferentiated mouse retina. **A** shows double-labeling of mouse retina at stage P3 with antibodies against mPins and Numb proteins. The first and second panels from the left correspond to labeling for mPins (red) and Numb (green), respectively. The third panel represents the merged image. The fourth panel shows the colocalization area between mPins and Numb (white). **B** shows a scatter plot of red versus green intensities generated by Imaris software. The percentage of material colocalized and the Pearson coefficient are indicated below the graph. At P3, the 2 antibodies gave almost identical staining patterns, and colocalization (fourth panel, white) seemed to be more intense in the ganglion cell layer (GCL), the inner part of the neuroblastic layer (Nbl), and the retinal pigment epithelium (RPE; **A**). The colocalization quantification value supports our observations. We obtained a large percentage of material colocalized (about 40%) and a high Pearson coefficient for colocalized voxels (0.73; **B**).

We investigated the involvement of mPins protein in ACD mechanisms during mouse retinal development by immunocolabeling the mPins and Numb proteins, using Numb as a marker of ACD (Figure 11A). We assessed the extent of colocalization of these 2 labels, using the colocalization module of Imaris software to analyze whole stacks of confocal sections, as described by Costes and Lockett [39]. The colocalization measurements used were “percentage of material colocalized” and the “Pearson correlation coefficient.” At the P3 stage, immunostaining for mPins and Numb was colocalized throughout the neuroblastic layer and in the ganglion cell layer (white; Figure 11A). This colocalization appeared to be more intense in the ganglion cell layer and in the inner part of the neuroblastic layer. As expected, the colocalization quantification value supported our observations. Indeed a high percentage of colocalized material was observed (about 40%), together with a high Pearson coefficient in colocalized voxels (0.73; Figure 11B).

### Variation of mPins levels in the lens

We report that the distribution of mPins immunostaining was consistent with the distribution of *mPins* mRNA in the lens during late embryonic and late postnatal stages. By contrast, at the P3 and P5 stages, we clearly observed *mPins* mRNA in the developing lens, whereas the immunolabeling obtained at this stage was diffuse and provided no clear evidence of the presence of the protein in cells. We performed RT–PCR and western blotting to quantify mPins mRNA and protein levels in the lens at birth and throughout postnatal development to clarify this point.

**Figure 12:**
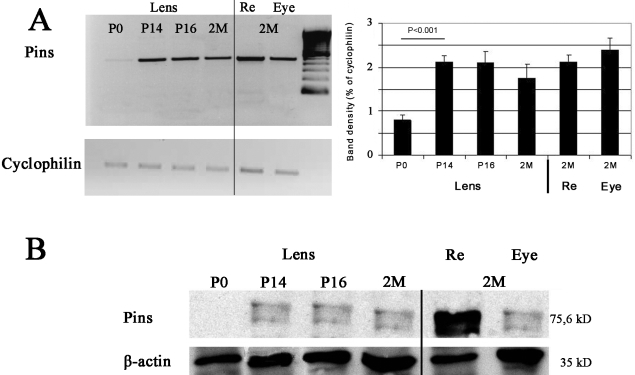
*mPins* mRNA and protein levels in the mouse lens during postnatal development and adulthood. **A** shows semiquantitative RT–PCR results for determination of the relative amounts of *mPins* mRNA in the lens at P0, P14, P16, and 2 months after birth (2 M) and their comparison with the relative amounts in the retina and whole eye at 2M. The 473 bp and 311 bp bands correspond to the *mPins* and *cyclophilin* PCR products, respectively. The relative levels of *mPins* mRNA are also calculated as a ratio of the intensity of the *mPins* band to that of the *cyclophilin* band. The densitometric analysis of PCR band intensities shows low levels of *mPins* mRNA in the lens at the P0 stage. These levels increase significantly at P14 and are maintained until adulthood. They are similar to those in the adult retina and whole eye. **B** shows a western blot used to determine the relative levels of mPins protein in the lens at P0, P14, P16, and 2M; these levels were compared with those in the retina and whole eye at 2M. Specific bands for mPins (75.6 kDa) and β-actin (35 kDa) are detected in the lens, whole eye, and neuroretina extracts. The mPins protein is not detected in the lens at the P0 stage. However, it is detected at the P14 stage, and the levels are maintained from this stage to adulthood. Retina and whole eye at 2M are used as positive controls. The western blot images in Panel B are indeed cropped. The lanes are of course unambiguously otherwise free of signal.

The product of PCR amplification with *mPins*-specific primers gave a clear band of the expected size (473 bp). An analysis of PCR band intensity showed that *mPins* mRNA level was very low in the lens at the earliest postnatal stage considered, P0 (p<0.001). *mPins* RNA levels in the lens increased during postnatal development, until P14, remaining stable thereafter into adulthood. The levels observed at P14 were similar to those in the adult retina and whole eye (Figure 12A).

We then performed western blot analysis with anti-Pins antibodies, to confirm these observations and the production of the mPins protein in the lens at the corresponding postnatal and adult stages. The purified antibody provided by Xiaohang Yang recognized a 75 kDa protein in all protein extracts, consistent with the predicted molecular weight of the mouse Pins protein. However, an additional band was recognized, corresponding to the LGN homolog, AGS3. An antibody against β-actin was used as an internal control to ensure equal protein loading. Retina and whole eye at 2M were used as positive controls. The results obtained were consistent with RT–PCR data. No significant amount of mPins protein was detected by western blot analysis at the earliest postnatal stage, P0, but this protein was detected at P14. The levels of mPins protein detected remained constant throughout adulthood (Figure 12B). Thus, both RT–PCR and western blotting results indicated that the lens contained small amounts of mPins at P0, with significantly higher levels observed at P14 and maintained throughout adulthood (Figure 12A,B). For the validation of these results, we performed the same western blot analysis with 2 different anti-Pins antibodies, as used in immunohistochemistry. Each purified antibody specifically recognized a band of 75 kDa in all protein extracts tested, consistent with the predicted molecular weight of the mouse mPins protein. Identical results were obtained with all three antibodies (data not shown).

## Discussion

The nervous systems of vertebrates and invertebrates display tremendous diversity in cell types. In vertebrates, neurogenesis is thought to involve the proliferation of progenitor cells by symmetric division, followed by several asymmetric divisions giving rise to different populations of neurons and glia. The generation of daughter cells with different cell fates involves 3 levels of control: 1) regulated programs of gene expression, controlling cell fate; 2) unequally segregated fate determinants, which, in turn, regulate programs of gene expression in only one of the two daughter cells; and 3) cell polarity machinery coordinating the asymmetric localization of fate determinants with respect to the cell division plane. Many genes essential to this process have been identified, including those encoding Bazooka/Par3, Insc, and Pins, which plays an important role in the segregation of cell-fate determinants and rotation of the spindle [6,10]. In this study, we investigated mPins mRNA and protein expressions in the developing mouse whole eye and, more specifically, in the retina, in which ACD has been reported in rat [31,32] and chicken [33].

Late embryonic mouse retina (E11.5 and E18.5) displayed strong *mPins* hybridization signals in all cells of the neuroblastic layer which are on different stages of proliferation as well as differentiation. *mPins* mRNA was absent from the GCL, which is the first layer to differentiate. At these developmental stages, cell divisions may occur in diverse progenitor cells, which are still endowed with high mitotic potentials, and in many progenitor cells already engaged in a cell fate pathway but retaining some mitotic capability. The mouse retina continues to develop after birth. It is therefore not surprising that from P3, the cells of the inner neuroblastic layer continue to display strong mPins immunoreactivity as the retinal progenitors in this layer continue to undergo cell division. These results are consistent with those reported for the developing mouse CNS [19], in which mouse *Pins* gene expression has been shown to be limited to zones of proliferation and absent from differentiating postmitotic cells. By contrast with embryonic stages, at these early postnatal stages, *mPins* mRNA was detected in the GCL. The pattern observed for mPins protein in immunohistochemistry experiments could be readily reconciled with that observed for the distribution of *mPins* mRNA determined by in situ hybridization. It is noteworthy that mPins mRNA and protein seemed to be stronger in the inner cells of the neuroblastic layer at early postnatal retinal developmental stages.

Based on this pattern of *mPins* expression in dividing cells and the demonstration that mPins is a functional homolog of *Drosophila* Pins that can substitute for Pins function in *Drosophila* neuroblasts, our results strongly suggest that mPins may be involved in ACD in the developing mouse retina. The retinal neuroblastic layer undergoes cell division, and ACD has been shown to occur in the retina. These data suggest that mPins may be involved in ACD in the developing retina. Such a role would require mPins to have the same cellular distribution as a marker of ACD, such as Numb. The results of the qualitative and quantitative analysis of colocalization between mPins and Numb in an undifferentiated retina at an early postnatal stage (P3) suggest that this is the case. The percentage of material colocalized and the Pearson correlation coefficient must be considered together to evaluate the extent of colocalization. The “percentage of material colocalized” (40%) obtained for the developing retina indicated strong, widespread colocalization of the 2 proteins. Moreover, the Pearson coefficient (0.73) obtained at P3 showed that the intensities of the proteins studied varied together. Thus, we have shown that *mPins* is widely expressed in the retinal neuroblastic layer and that mPins protein is colocalized with a marker of ACD in these dividing cells. Overall, our results strongly suggest the involvement of mPins in ACD in the developing retina.

However, previous studies in mice involving the analysis of marked clones in adult retinas have suggested that retinal cells do not follow a stem cell mode of division. Instead, both daughter cells of a progenitor may continue to divide. These studies have even suggested that cell type determination in the rodent retina is independent of lineage [40]. These findings are debatable, because the authors used only clone size as the crucial parameter for interpretation of their results. However, it has long been known that at least 2 types of neurons and Müller glia in postnatal rodent retina may arise from a common progenitor [40]. Nevertheless, it seems likely that mPins is involved in ACD in the retina, because ACD has been reported in the retina in both rat [31] and chicken [33]. In addition, Insc, which acts together with Pins in ACD, has been shown to regulate spindle orientation and cell fate in the developing retina—2 major mechanisms involved in ACD [21].

So, how does mPins regulate ACD in the retina? The orientation of cell division is important for invertebrate development, but it has been suggested that vertebrates develop in a different manner entirely dependent on cell migration and diffusible morphogens [41]. Studies have demonstrated correlations between spindle orientation and the fate of the resulting daughter cells [21,42]. In the developing mouse brain [43] and in rat retina [32], progenitor divisions along the vertical axis are more likely to generate 2 different daughter cells, whereas parallel divisions are usually symmetric. Mammalian Insc regulates the orientation of neural progenitor divisions, playing an important role in specifying cell fate in the retina and determining whether divisions are symmetric or asymmetric [21]. Insc may act by recruiting the Pins homolog, LGN, to the apical cell cortex, as described in *Drosophila*. In addition, LGN may activate G proteins regulating the attachment of astral microtubules to the cell cortex in mouse [19]. These mechanisms would result in polarized G protein activation. Moreover, LGN binds the nuclear mitotic apparatus protein NuMA, which is a large coiled-coil, and microtubule-binding protein, which organizes the orientation of the spindle poles. NuMA/LGN interaction is required for the binding of LGN to the α subunits of G-protein (Gα) in the cell cortex. Thus, the NuMA/LGN/Gα complex regulates the interaction of aster microtubules with the cell cortex, causing chromosome segregation during mitosis [44]. Furthermore, many of the proteins involved in ACD in *Drosophila* have orthologs in mammals, and the asymmetric distribution of these compounds is conserved in various mammalian tissues. For example, mammalian Numb is asymmetrically distributed at the apical pole of dividing neuroepithelial cells in the developing mouse cortex [36], in the rat retina [31,36], and in zebrafish retina [45]. Based on our results demonstrating the colocalization of mPins and Numb and the relative conservation of interactions between proteins involved in ACD from *Drosophila* to vertebrates, there is growing evidence that mPins may regulate ACD by the described mechanisms.

One of the major findings of this study is the demonstration that *mPins* is expressed in the postnatal terminally differentiating retinal neurons. The cell bodies of the ganglion cell layer are immunolabeled for mPins from P3. Moreover, at later postnatal stages, *mPins* mRNA and protein are detected in all layers of the retina other than the plexiform layers. No significant amount of *mPins* mRNA was detected in the plexiform layers, whereas these layers did contain the mPins protein. The mPins protein may therefore be produced in neuronal cell bodies and translocated into neuronal processes and terminals. The same pattern of *mPins* expression was observed in the adult retina. Weak immunolabeling in the outer plexiform layer and strong labeling in the photoreceptor inner segments were also observed. If the major role of mPins is related to the orientation of the mitotic spindle during retinal development, what role does this protein play in the quiescent nondividing neuronal cells of the retina? Like many molecules, Pins seems to perform different functions in the retina during development and in adulthood. There are two possible explanations for these different roles: 1) Pins may be a multifunctional protein; or 2) Pins may interact with different partners during these two different phases of mammalian life. Previous studies reported a crucial role of Pins in differentiated neurons [45]. In mammals, Pins and Gαi, form a protein complex with SAP102 and NMDAR in hippocampal neurons. The formation of this complex has been shown to be essential for NMDAR translocation to the plasma membrane and its incorporation into the postsynaptic membrane of dendritic spines [46]. Interestingly, both rods and cones use the excitatory amino acid glutamate to transmit signals to the second-order neurons (retinal bipolar neurons) in the chain. Retinal ganglion cells and some amacrine cell types express functional NMDA receptors in addition to non NMDA receptors [47–52]. We suggest that mPins may regulate vesicle trafficking in retinal neurons, as described in hippocampal neurons [45]. However, further experiments are required to confirm this hypothesis. How can we reconcile the roles of mPins in processes as diverse as spindle orientation and vesicle trafficking? During spindle orientation, Pins may mediate microtubule attachment to the plasma membrane by interacting with microtubule-associated protein (NuMA) and Gαi. Pins may also act in a similar manner in vesicle trafficking, mediating the attachment of NMDAR-containing vesicles to microtubule motors, thereby facilitating their transport to the plasma membrane. mPins protein was detected on both sides of the inner nuclear layer, in areas potentially corresponding to the horizontal and amacrine cells. However, double labeling with specific markers of amacrine or horizontal cells is required to confirm this hypothesis. These cells are involved in the transmission of electrical messages in visual signals. Indeed, the horizontal cells establish direct connections with the photoreceptor, whereas the amacrine cells are responsible for lateral modulation of the vertical transmission channel of the signal, establishing synapses with and between bipolar cells. In retinal horizontal and bipolar cells, neuronal signaling and membrane excitability are mediated principally by G-protein-coupled Kir3 (GIRK) channels. Various subunits of Kir channels have been characterized in rat retinal ganglion cells [53]. GIRK (G protein-activated inwardly rectifying potassium) channels are Gβγ effectors activated by inhibitory transmitters to dampen excitatory synaptic transmission and reduce the excitability of central neurons [54–56]. In mammals, LGN has been shown to play an essential role in maintaining basal GIRK channel activity and harnessing neuronal excitability [57]. In light of these roles in neurons, the strong expression of mPins in the adult mouse neural retina and the many possible roles of the mPins protein in cellular trafficking and neuronal excitability within the retina appear much less surprising.

The mPins mRNA and protein are observed in the RPE during early postnatal development, but also in adult RPE. The RPE constitutes the outer hemato-ocular barrier, and the cells constituting the RPE cell layer are strongly linked by tight junctions. In mammalian epithelia, PAR-3 localizes to tight junctions at the apical/lateral boundary [58], and is involved in the assembly of these junctions [59]. Human Insc establishes an interaction between LGN and PAR-3 [60]. It would be interesting to assess the involvement of mPins in epithelial tight junction formation and the maintenance of the structure of these junctions during adulthood, in the RPE, and in the ciliary and corneal endothelium, by appropriate experiments.

The corneal epithelial cells—particularly the basal epithelial cells—express large amounts of mPins mRNA and protein. It is currently widely thought that the renewal of this epithelium requires the production of new cells through mitotic activity in the limbal basal cell layer, these cells then displacing the existing cells both superficially and centripetally. Francois Majo (Hôpital ophtalmique universitaire Jules-Gonin, Lausanne, Switzerland; unpublished data, personal communication) has proposed a model in which corneal epithelial cell generation favors horizontal symmetric cell divisions, ensuring the self-renewal of basal corneal epithelial progenitor cells, and asymmetric vertical cell divisions, generating both basal corneal epithelial cells and differentiating suprabasal cells moving toward the inner part of the tear film at the surface of the cornea. This model is consistent with results obtained for the epidermis [61]. The same mechanism of spindle reorientation may therefore lead to the specification of different cell types in the developing retina, and stratification in the epidermis and cornea. Based on these data, we suggest that mPins may also be involved in corneal cell division.

One of the major findings of our study is the different patterns of mPins mRNA and protein distribution in the lens during development and in adulthood, with protein levels in the lens increasing during postnatal development. At the E14.5 stage, mPins protein is produced in the anterior region of the lens corresponding to the lens anterior epithelial cells. However, no significant mRNA hybridization signal was detected in this region of the lens at this stage. The level of *mPins* mRNA may have been just below the detection threshold of the method used, such that hybridization signals were not considered significant. This experimental artifact may account for the lack of detection of *mPins* mRNA in the lens at this stage. Interestingly, the mPins mRNA and protein are significantly detected, at late stages, not in the anterior, but in the posterior part of lens, corresponding to the formation of differentiated secondary fibers. From P9, mPins mRNA and protein were detected unambiguously in the anterior epithelial cells, the proliferating cells of the germinative zone, cells of the transitional zone and differentiated lens fibers at postnatal stages. By contrast, at the earliest stages of postnatal lens development (P3 and P5), the immunostaining observed in the lens was diffuse and appeared less significant. However, by RT–PCR we detected significant amounts of mPins in the lens from P0. During embryonic development, the germinative zone is already formed and the lens undergoes rapid growth. These findings raise two questions: 1) How can we interpret the finding that mPins expression becomes detectable in the mitotic germinative zone only during later postnatal stages? and 2) How can we explain the changes in mPins protein profile during development? It has been shown that Dlg protein may be required for the maintenance of cell cycle control in the lens [62]. In *Drosophila,* the interaction between the proteins Pins and Dlg has been shown to regulate cell division [16]. Interestingly, the pattern of Dlg expression in the lens reported by Nguyen [63] is similar to the pattern of mPins expression reported here. Yeast two-hybrid screening with LGN/mPins had identified Dlg as an interacting protein (Blumer J. B. Department of Cell and Molecular Pharmacology, Medical University of South Carolina, Charleston, SC, unpublished data). Interactions between mPins and Dlg may be involved in cell division and proliferation in the mouse lens. Given the role of mPins in cell proliferation processes, it is surprising that mPins is detected in the germinative zone only after birth. Moreover, mPins levels in the lens increase during postnatal development. We can therefore speculate that this increase in mPins levels during postnatal development is correlated with lens growth until the lens reaches its adult size. The spatial distribution and time course of mPins expression in the lens reported here suggest that mPins protein expression is strongly controlled, and that mPins functions are thus important throughout lens development. However, the roles of this protein remain unclear, and further experiments are required to determine these roles.

In conclusion, we have determined the pattern of *mPins* gene expression and mPins protein distribution in mouse eye from embryonic to adulthood. Our results suggest that mPins may be involved not only in proliferation, but also in differentiation and the maintenance of differentiation in mouse eye. These findings highlight the importance of further exploration of the role of mPins and of the major sets of polarity genes during eye development, normal ocular functioning in adulthood and eye aging.
